# Comparative Effectiveness of Different Probiotic Delivery Methods in Oral Candidiasis: A Systematic Review

**DOI:** 10.3390/microorganisms13122883

**Published:** 2025-12-18

**Authors:** Reihaneh Ashouritoustani, Cláudia Pinho, Ana Isabel Oliveira, Piedade Barros, Agostinho Cruz

**Affiliations:** 1 ESS, Polytechnic of Porto, Rua Dr. António Bernardino de Almeida 400, 4200-072 Porto, Portugal; 10240966@ess.ipp.pt; 2 REQUIMTE/LAQV, ESS, Polytechnic of Porto, Rua Dr. António Bernardino de Almeida 400, 4200-072 Porto, Portugal; clp@ess.ipp.pt (C.P.); aio@ess.ipp.pt (A.I.O.); pbarros@ess.ipp.pt (P.B.)

**Keywords:** oral candidiasis, Probiotic, microbiota, delivery methods, fungal infections, efficacy, *Candida*, antifungal

## Abstract

Oral candidiasis, mainly from *Candida albicans*, affects immunocompromised individuals, the elderly, and denture wearers. Probiotics offer immunomodulatory and microbiota-balancing benefits as potential antifungal alternatives. However, the comparative impact of different probiotic delivery methods remains inadequately explored. This systematic review evaluated the effectiveness of various probiotic delivery methods in reducing *Candida* colonization and clinical symptoms in oral candidiasis. Following PRISMA 2020 guidelines, a systematic review search across multiple databases included human clinical studies based (Medline, Web of Science, ScienceDirect, and ProQuest) on PICO criteria across all age groups. Outcomes assessed included *Candida* load, oral microbiota changes, symptom improvement, and disease recurrence. Of 297 articles screened, 10 met inclusion criteria. Delivery methods investigated included lozenges, capsules, yogurt, and cheese. Most studies reported reductions in *Candida* colony-forming units (CFUs) or prevalence, mainly for *C. albicans* and for non-albicans species, with probiotics such as *Lactobacillus reuteri*, *L. rhamnosus*, *L. acidophilus*, and *Bifidobacterium* strains. Some studies reported improved immunological markers, while symptom relief, especially when probiotics were combined with antifungals. Probiotics reduce *Candida* colonization and symptoms, with potential prolonged effects. They show promise as adjunctive therapies, but standardized, large-scale trials are needed for optimization.

## 1. Introduction

Oral candidiasis, primarily attributed to the opportunistic pathogen *Candida albicans*, is a common fungal infection affecting the oral mucosa, particularly in immunocompromised individuals [1], the elderly [2], denture wearers [3,4,5], and patients undergoing chemotherapy or radiotherapy [6]. *Candida albicans* is part of the normal microbiota but may become pathogenic under compromised host conditions, contributing to biofilm formation and resistance to antifungal agents [7].

The infection ranges from asymptomatic colonization to overt mucosal lesions, often exacerbated by factors such as xerostomia [8], diabetes [9], poor oral hygiene, and poorly adapted dentures [10,11]. Dentures themselves serve as reservoirs for *Candida* spp. due to their porous nature and susceptibility to biofilm accumulation [12,13].

While conventional antifungal therapies such as nystatin and azoles remain the standard of care [14,15], their efficacy is often limited by side effects [16,17], and the emergence of drug-resistant *Candida* strains [18]. Consequently, there is growing interest in alternative and complementary therapies that can modulate the host’s microbiota and immune response with fewer adverse effects [19,20,21].

Probiotics—defined as “live microorganisms which, when administered in adequate amounts, confer a health benefit on the host” [22]—have gained attention as supportive or alternative therapies for *Candida*-associated oral infections. Certain *Lactobacillus* and *Streptococcus* strains exhibit antifungal effects through competitive inhibition [23], production of organic acids [24], hydrogen peroxide, and bacteriocin-like substances, which suppress *C. albicans* growth and biofilm formation [25]. Moreover, probiotics can modulate host immunity by stimulating cytokine release and enhancing macrophage and T-cell activity, thereby improving mucosal defence [26]. These combined antimicrobial and immunomodulatory effects make probiotics a promising adjunctive approach for managing oral candidiasis [21,26].

Probiotic strains such as *Lactobacillus rhamnosus*, *L. acidophilus*, *L. reuteri*, and *Streptococcus salivarius* K12 have demonstrated antifungal activity in both in vitro [23], and in vivo studies [19,27]. Additionally, specific strains like *L. rhamnosus* GG and *L. reuteri* have shown effectiveness when delivered in cheese or lozenge forms [19,28].

While numerous studies indicate that probiotics can help lower oral *Candida* spp. Levels [20,21,29], there is a lack of comparative research on the effectiveness of different delivery methods [30]. Only a few investigations have directly assessed forms such as lozenges, cheese, yogurt, or capsules in terms of clinical efficacy, patient adherence, and long-term impact [20,21,30]. Delivery method influences probiotic retention time in the oral cavity, patient adherence, and overall therapeutic effectiveness, making it a clinically relevant factor [28]. This underscores the need for a systematic comparison to inform optimal probiotic strategies for managing oral candidiasis.

This systematic review aims to critically assess and compare the effectiveness of various probiotic delivery methods in reducing *Candida* spp. colonization and clinical symptoms associated with oral candidiasis. By synthesizing evidence from diverse clinical settings and populations, this review seeks to identify the most promising delivery formats and inform future therapeutic strategies for managing this prevalent oral infection.

## 2. Materials and Methods

This systematic review was conducted in accordance with the PRISMA 2020 (Preferred Reporting Items for Systematic Reviews and Meta-Analyses) guidelines [31]. The protocol of this systematic review was registered in the PROSPERO database [32] (CRD420251060214).

### 2.1. Focused Question

The focused question was “How do different probiotic delivery forms influence the effectiveness of probiotics in the treatment of oral candidiasis in human clinical trials?”

### 2.2. Patients, Interventions, Control, Outcome (PICO)

The study follows the Patient-Intervention-Comparison-Outcome (PICO) framework to ensure a well-defined research question. In this review, the population (P) consists of patients diagnosed with oral candidiasis. The intervention (I) involves the use of different probiotic delivery methods, while the comparison (C) involves different probiotic delivery forms. (It should be noted that not all included studies directly compared delivery methods within the same trial. Comparative analysis was therefore conducted qualitatively across studies). The outcome (O) focuses on the effectiveness in reducing *Candida* colonization and symptom relief [33].

### 2.3. Eligibility Criteria

The inclusion criteria were: (a) studies conducted on participants with either clinically confirmed oral candidiasis or microbiologically confirmed *Candida* colonization without clinical signs, (b) participants of all age groups including children, adults, and the elderly, (c) inclusion of individuals with or without denture use, (d) clinical studies involving human subjects (e.g., randomized clinical trials), (e) studies in which probiotics were used as the primary intervention, (f) probiotics administered in any form with reported dosage, treatment duration, and method of administration, (g) studies reporting at least one of the following outcomes: reduction in *Candida* colonization load, changes in oral microbiota balance, reduction in the severity of clinical symptoms, or recurrence rate of the disease during follow up, (h) articles published in English, and (i) no restriction on the year of publication.

Studies were excluded if they met one or more of the following conditions: (a) focused on fungal infections other than oral candidiasis (e.g., vaginal, cutaneous, or systemic candidiasis); (b) did not use probiotics as the primary intervention for treatment; (c) included participants presenting with clinical denture stomatitis; (d) involved individuals with cancer (The exclusion of cancer patients refers to individuals with an active cancer, including those undergoing or who have recently completed chemotherapy or radiotherapy. These conditions substantially alter oral immunity and could introduce heterogeneity in treatment response. Immunocompromised individuals without cancer (e.g., HIV-infected participants) were not excluded. None of the included studies involved participants with cancer.); (e) were published in languages other than English; (f) were of the following study types: in vitro (laboratory) studies, animal studies, systematic reviews or meta-analyses, and studies with incomplete data (e.g., lacking efficacy outcome reports).

### 2.4. Search Strategy

A comprehensive search was carried out in accordance with PRISMA guidelines, using electronic databases such as Medline, Web of Science, ScienceDirect, and ProQuest on 26 February 2025. The search terms included “probiotic,” “microbiota,” “oral candidiasis,” “administration,” and “use,” which were applied in various ways and adapted for each database using appropriate indexing terms and Boolean operators. The search strategies were as follows:

• MEDLINE: (((probiotic[MeSH Terms]) OR (microbiota[MeSH Terms])) AND (oral candidiasis[MeSH Terms])) AND ((administration) OR (use))
• Web of Science Core Collection: (((TS=(Probiotic)) OR TS=(Microbiota)) AND TS=(Oral Candidiasis)) AND (TS=(Use) OR TS=(Administration)), with the “Article” document type filter applied.
• ScienceDirect: ((“probiotic” OR “microbiota”) AND “oral candidiasis” AND (“administration” OR “use”)), with the “Research Articles” filter applied.
• ProQuest: noft(probiotic OR microbiota) AND noft(oral candidiasis) AND noft(administration OR use), using the “Anywhere except full text—NOFT” field and applying the “Scholarly Journals” and “Article” filters.

### 2.5. Data Extraction and Quality/Risk of Bias Assessment

Study eligibility was assessed independently by two authors based on the title and abstract of the articles. Following this selection, a full-text review of the selected articles was conducted by two authors in order to confirm whether they met the inclusion criteria. In case of disagreements about the selection of studies, decisions were made through discussion with another researcher.

The methodological quality and risk of bias in the included studies were assessed independently by two authors using the JBI critical appraisal checklist [34,35]. Domain-specific judgments (e.g., randomization procedures, allocation concealment, blinding, follow-up completeness, and statistical analysis) were assessed according to the JBI checklist criteria. This study uses Robvis to create plots for JBI critical appraisal checklist [36].

### 2.6. Synthesis Methods

All included studies fulfilled the quality criteria. Due to heterogeneity in study designs, probiotic strains, and intervention protocols, a meta-analysis was not feasible. Therefore, a narrative synthesis approach was adopted. For this synthesis, studies were grouped and compared qualitatively based on their probiotic delivery method, study characteristics, and outcomes, which are structured in Table 1, Table 2 and Table 3.

## 3. Results

### 3.1. Study Selection

**Figure 1:**
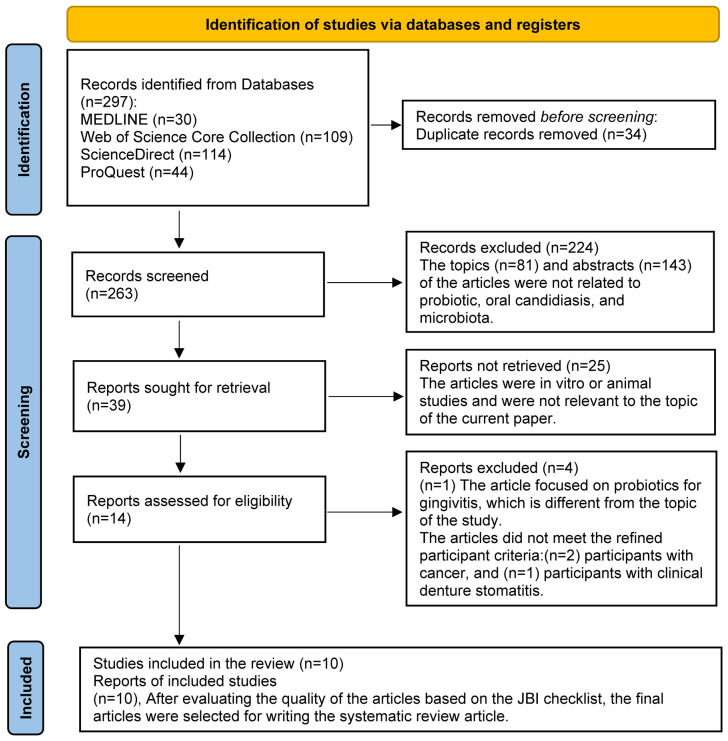
PRISMA 2020 flow diagram for new systematic reviews.

A total of 297 records were initially retrieved through database searches. After removing 34 duplicate entries, 263 unique records remained for screening. Following a two-stage screening process against eligibility criteria, 10 studies met the inclusion criteria and were selected for the final systematic review, based on a quality appraisal using the JBI checklist. The screening process was conducted using Rayyan [42], a web-based tool designed for systematic reviews. Also, no time limit was applied to extracting articles (Figure 1).

### 3.2. General Characteristics of the Included Studies

All studies were conducted in clinical or hospital-based settings between 2007 and 2024. The studies were reported to be conducted in Brazil, Egypt, Sweden, China, the USA, Turkey, and Finland. The inclusion of studies from these countries was not intentional or geographically restricted; rather, it resulted from the systematic screening process based on predefined inclusion and exclusion criteria. Articles meeting the eligibility criteria were included regardless of their country of origin. Seven of the studies were randomized controlled trials (RCTs) [3,19,20,37,39,40,41], while three were quasi-experimental studies [1,21,38], all involving human subjects. Trial registration numbers for several studies are presented in Table 1.

The mean age of participants varied widely across studies, ranging from young adults to elderly individuals. In most studies, the participants were older adults, often over 60 years of age, reflecting the focus on specific populations such as denture wearers, and frail elderly. The sample sizes of the included studies were highly heterogeneous, ranging from 24 to 276 participants across trials (Table 1).

### 3.3. Population Characteristics of the Included Studies

The 10 studies exhibited varied inclusion and exclusion criteria (Table 2). Inclusion criteria often specified oral conditions (e.g., *Candida* colonization, denture use) [3], specific populations (e.g., elderly, human immunodeficiency virus (HIV)-positive women) [1,21,41]. Exclusion criteria frequently involved use of antifungals/antibiotics [38,39], reported of consumption of probiotics [37], lactose intolerance [40], severe systemic diseases [20], and cognitive impairment [41].

### 3.4. Outcome Characteristics of Probiotic Interventions in Oral Candidiasis

The included studies, detailed in Table 3 for strain composition, CFU counts, and exact administration protocols, evaluated probiotic effectiveness. The findings are summarized below according to delivery method.

#### 3.4.1. Lozenges

Four studies administered probiotics via lozenges [20,37,39,41]. The lozenges contained various strains, such as including 7 *Lactobacillus* strains and 5 *Bifidobacterium* strains species [37], 2 *L. reuteri* strains (DSM 17938 and PTA 5289) [41], and *Streptococcus salivarius* K12 [39]. Treatment durations ranged from 4 weeks [20,39], 8 weeks [37], and 12 weeks [41], with administration frequencies of once daily [37], twice daily [39,41], or three times daily [20]. Reported outcomes included significant reductions in *Candida* CFU/mL compared with control groups (*p* < 0.001) [37] and assessments of clinical symptoms [20].

#### 3.4.2. Capsules

One study used a capsule containing *L. rhamnosus* HS111, *L. acidophilus* HS101, and *B. bifidum*, applied daily to the upper denture for five weeks [3]. The probiotic group showed a significantly lower *Candida* detection rate (16.7%) than the placebo group (92.0%) (*p* < 0.0001).

#### 3.4.3. Sachets

Two studies administered probiotics in sachet form, mixed with liquid before intake [21,38]. Treatment durations were 10 days [38] and 30 days [21], with daily [38] or thrice-weekly [21] dosing. Outcomes included reductions in *Candida* counts and measurement of salivary anti-*Candida* IgA levels [21].

#### 3.4.4. Probiotic Products

Three studies used probiotic products as delivery vehicles: two probiotic cheeses [19,40] and one probiotic yogurt [1]. Intervention durations were 30 days [1], 8 weeks [40], and 16 weeks [19], with daily consumption. Two studies reported significant reductions in *Candida* CFU/mL compared with controls [19,40], while one study found potential reductions but noted limitations due to a small sample size [1].

Outcome measurements across all included studies primarily focused on quantification of *Candida*, typically through culture on Sabouraud Dextrose Agar (SDA) followed by species identification [1,3,21,37].

### 3.5. Quality of the Included Studies

The methodological quality of the 10 included studies was assessed using the relevant JBI critical appraisal tools, selected based on each study’s design. Three studies were evaluated using the JBI Checklist for Quasi-Experimental Studies [34], while seven studies were assessed using the JBI Checklist for Randomized Controlled Trials [35]. In cases of disagreement, a third researcher was consulted to facilitate a consensus-based resolution.

For clinical trials, the quality assessment considered factors such as the adequacy of the randomization process, the representativeness of participant selection, the presence and length of the follow-up period (and any disadvantages associated with short follow-up), and the appropriateness of the control and intervention groups.

Each checklist includes four possible responses: “Yes,” “No,” “Unclear (UC),” and “Not Applicable (N/A).” A higher proportion of “UC” and “N/A” responses suggests either poor reporting or methodological weaknesses. Studies with substantial ambiguity in these areas were considered to be at a higher risk of bias. None of the included studies were excluded following the quality appraisal, as all were considered to meet the minimum methodological standards required for inclusion.

**Figure 2:**
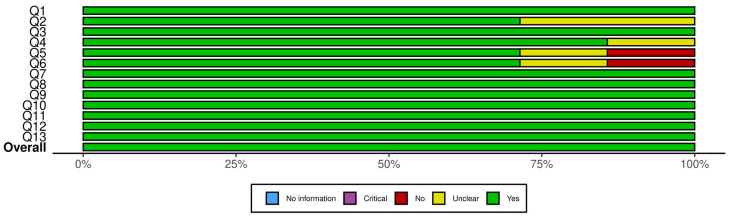
Summary plot for JBI critical appraisal checklist for randomized controlled trials. Q1. Was true randomization used for assignment of participants to treatment groups? Q2. Was allocation to treatment groups concealed? Q3. Were treatment groups similar at the baseline? Q4. Were participants blind to treatment assignment? Q5. Were those delivering treatment blind to treatment assignment? Q6. Were outcomes assessors blind to treatment assignment? Q7. Were treatment groups treated identically other than the intervention of interest? Q8. Was follow up complete and if not, were differences between groups in terms of their follow up adequately described and analyzed? Q9. Were participants analyzed in the groups to which they were randomized? Q10. Were outcomes measured in the same way for treatment groups? Q11. Were outcomes measured in a reliable way? Q12. Was appropriate statistical analysis used? Q13. Was the trial design appropriate, and any deviations from the standard RCT design (individual randomization, parallel groups) accounted for in the conduct and analysis of the trial?

**Figure 3:**
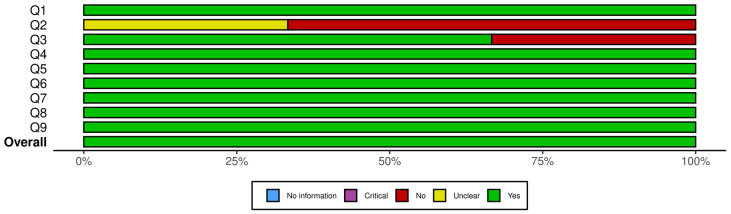
Summary plot for JBI checklist for quasi-experimental studies. Q1. Is it clear in the study what is the “cause” and what is the “effect” (i.e., there is no confusion about which variable comes first)? Q2. Was there a control group? Q3. Were participants included in any comparisons similar? Q4. Were the participants included in any comparisons receiving similar treatment/care, other than the exposure or intervention of interest? Q5. Were there multiple measurements of the outcome, both pre and post the intervention/exposure? Q6. Were the outcomes of participants included in any comparisons measured in the same way? Q7. Were outcomes measured in a reliable way? Q8. Was follow-up complete and if not, were differences between groups in terms of their follow-up adequately described and analyzed? Q9. Was appropriate statistical analysis used?

Figure 2 presents a summary plot of the JBI Critical Appraisal Checklist for Randomized Controlled Trials, and Figure 3 displays the corresponding plot for the JBI Checklist for Quasi-Experimental Studies. In these visual summary plots, “No information” is used in place of “N/A.”

A closer inspection of the RCTs showed that the highest sources of bias were related to allocation concealment and blinding. Specifically, two trials did not clearly report allocation concealment, and blinding procedures were either unclear or absent in one to two studies for participants, treatment providers, and outcome assessors. All other domains showed consistently strong reporting across the seven RCTs. For the quasi-experimental studies, uncertainty arose from the use and reporting of control groups: two studies did not clearly describe whether a control group existed, and one lacked a control group entirely. Baseline comparability was unclear in one study, but all other methodological domains were well reported. Overall, the areas with the most ambiguity involved blinding in RCTs and control-group structure in quasi-experimental designs. Despite these limitations, none of the studies exhibited risk levels sufficient to warrant exclusion.

A comprehensive summary of the quality appraisal results, including detailed tables, is available in the Supplementary Materials.

### 3.6. Results Synthesis

The narrative synthesis of the 10 studies is presented in Table 1, Table 2 and Table 3, with risk-of-bias assessments illustrated in Figure 2 and Figure 3. The synthesis also highlights gaps in the available evidence.

## 4. Discussion

This systematic review investigated how different probiotic delivery forms influence the effectiveness of probiotics in treating oral candidiasis in humans. The 10 included studies used delivery methods that varied substantially, including capsules, lozenges, sachets, yogurt, and cheese. Probiotics, whether used alone or as adjuvants to conventional antifungal therapies, were generally effective in reducing oral *Candida* colonization.

The importance of probiotic delivery systems in oral health has received increasing attention, as probiotic efficacy depends not only on strain selection but also on the ability of the microorganisms to remain viable, and exert localized effects, although such mechanisms were not directly evaluated in the included studies. The delivery method is likely to influence probiotic performance; however, none of the included studies directly measured mucosal retention, survival, or adhesion, so these mechanisms remain theoretical and based on broader probiotic literature. Formulations that potentially prolong contact with the oral mucosa, such as lozenges, may provide enhanced local exposure. These mechanisms are theoretically relevant in the management of oral candidiasis, where sustained mucosal presence is essential to disrupt *Candida* biofilms and restore microbial balance. Additionally, the use of indigenous oral strains may enhance colonization efficiency and therapeutic efficacy, underscoring the importance of optimizing both the microbial composition and the delivery vehicle of oral probiotic products [43].

Lozenges were among the most consistently effective delivery forms [20,37,39,41]. Their mode of administration may provide longer oral exposure, which could contribute to clinical effects, although this was not measured in the included trials. In several studies, lozenges containing strains such as *Lactobacillus reuteri* [41], *Streptococcus salivarius* K12 [39], and mixed *Lactobacillus-Bifidobacterium* formulations [37] reduced *Candida* counts and, in some cases, shortened treatment durations when used as adjuncts to antifungal agents [39]. Slow dissolution may plausibly increase oral exposure time, but interaction with mucosal biofilms was not assessed in the included studies.

Capsule-based probiotics, when opened and applied directly to dentures or mucosal surfaces, have demonstrated significant efficacy in reducing oral *Candida* colonization in asymptomatic elderly denture wearers. This targeted application, involving the placement of the probiotic formulation on the maxillary denture in close contact with the palatal mucosa, may enhance localized probiotic activity and promote effective colonization at the mucosa-prosthesis interface [3].

Sachets dissolved in liquids showed significant antifungal and antimicrobial effectiveness, consistent with clinical findings demonstrating reduced microbial counts in oral regions and denture surfaces after probiotic intake. Their use also appeared to stimulate mucosal immune function, with studies reporting an increase in salivary anti-*Candida* IgA levels in elderly individuals, indicating enhanced secretory immune response and reinforcing their potential role in the management of oral candidiasis [21,38]. These findings suggest potential immunomodulatory effects, although causality cannot be established from the available evidence.

Probiotic products such as yogurt and cheese offered variable results. While two of the studies reported significant reductions in *Candida* colonization, another noted more limited or inconsistent effects. These variable outcomes may be partially related to limited oral contact inherent to food-based products, although this was not assessed in the included studies. Probiotic products, although easy to administer, may be less suitable for patients requiring sustained mucosal exposure to probiotics [1,19,40]. Nonetheless, probiotic dairy products may still play a supportive role, particularly in improving general microbial balance and contributing to immune modulation [44].

However, recent literature highlights that, although their mucosal contact is limited, fermented dairy products remain effective systemic probiotic carriers due to their capacity to maintain microbial viability during storage and gastrointestinal transit. Technological advances, such as microencapsulation and the use of exopolysaccharide-producing strains, have further enhanced the stability and functional efficacy of these products. Combined with broad consumer acceptance, these advances suggest that dairy matrices may function effectively as delivery vehicles, though the present review did not assess systemic outcomes, and such interpretations fall outside its scope [45].

## 5. Limitations

This review has some limitations. The included studies varied in design, participant characteristics, probiotic strains, dosage, sample sizes, and intervention duration. *Candida* quantification methods were not standardized across studies, and follow-up durations were often short, reducing the ability to assess long-term effects or recurrence. Although no geographic restrictions were applied during study selection, the distribution of available studies was uneven across regions, which may influence the generalizability of the findings.

Another limitation is that none of the included studies directly compared different probiotic delivery forms within the same trial, preventing definitive conclusions regarding the relative effectiveness of the various delivery systems.

The decision to exclude studies involving participants with active cancer was made because cancer therapies substantially alter oral immunity and could introduce clinically incomparable and heterogeneous treatment responses. While this criterion ensured greater homogeneity among the included populations, it also means that the findings primarily apply to non-cancer populations.

## 6. Conclusions

This systematic review suggests that probiotic delivery form may influence clinical outcomes in oral candidiasis; however, the available evidence remains limited and heterogeneous. Several studies reported favorable effects, particularly with delivery forms that potentially increase oral contact time, such as lozenges or formulations applied to dentures.

At the same time, orally ingested carriers such as dairy-based products also demonstrate promising findings. While their oral contact time is short, they remain practical options, especially when ease of use or integration into daily habits is important. However, evidence regarding systemic benefits or gut–oral interactions was beyond the scope of the included studies and requires further investigation.

Overall, current evidence supports the potential role of probiotics as adjunctive or preventive interventions for reducing *Candida* burden, but conclusions regarding the relative effectiveness of specific delivery methods remain preliminary. The small number of trials, variation in probiotic strains and dosages, and differences in *Candida* quantification methods limit the strength of comparative interpretations.

Future research should include well-designed, adequately powered randomized controlled trials that: directly compare different delivery vehicles under standardized conditions; use consistent probiotic strains or provide detailed strain-level reporting; apply uniform and validated *Candida* quantification methods; and incorporate longer follow-up periods to evaluate persistence of effects and recurrence.

In conclusion, while probiotic delivery form may influence therapeutic outcomes in oral candidiasis, stronger and more standardized clinical evidence is required before definitive recommendations can be made.

## Figures and Tables

**Table 1:** General characteristics of the included studies.

Year	Research Location	NCT Number *	Study Design	Participants Count	Mean Age of the Participants	Refs.
2024	Alexandria University, Egypt	NCT05358743	RCT	50	Placebo = 61.44 years ± 9.70, Probiotics = 60.20 years ± 9.81	Elsayes et al. [37]
2024	Usak University Faculty of Dentistry, Turkey	_____	QUASI-EXPERIMENTAL	61	66 years ± 7.6	Evirgen et al. [38]
2019	Peking University School and Hospital of Stomatology, China	ChiCTR-TCR-14005090 The study was registered with the Chinese Clinical Trial Registry (ChiCTR)	RCT	56	Control = 66.19 years ± 12.081, Probiotics = 61.15 years ± 10.227	Hu et al. [39]
2017	University of São Paulo, Brazil	RBR-5hn7gn The clinical trial was registered on a Brazilian registry: www.ensaiosclinicos.gov.br (accessed on 12 April 2025)	RCT	60	Group T1 = 61.7 years ± 14.1, Group T2 = 66.1 years ± 11.6, Group Control = 65.5 years ± 10.5	Miyazima et al. [40]
2015	University of São Paulo, Brazil	_____	RCT	59	61.6 years ± 9.8	Ishikawa et al. [3]
2015	nursing homes in the southern parts of Sweden	NCT02391532	RCT	215	Placebo = 87.7 years ± 7.7, Probiotics = 88.3 years ± 5.7	Kraft-Bodi et al. [41]
2014	West China College of Stomatology, Sichuan University, China	_____	RCT	65	Control = 64.84 years ± 11.92, Probiotics = 62.72 years ± 9.57	Li et al. [20]
2013	Georgetown University Medical Center, Washington, DC, USA	_____	QUASI-EXPERIMENTAL	24	+13 years	Hu et al. [1]
2012	city of Taubaté, SP, Brazil	_____	QUASI-EXPERIMENTAL	42	+65 years	Mendonça et al. [21]
2007	Helsinki area of Finland	_____	RCT	276	Control = 79.2 years, Probiotics = 78.9 years	Hatakka et al. [19]

* NCT Number (ClinicalTrials.gov Identifier): This unique identifier is issued for Randomized Controlled Trials (RCTs) upon registration in ClinicalTrials.gov and provides public access to the study’s aims, methods, and results, thereby promoting research transparency.

**Table 2:** Population characteristics of the included studies.

Include Criteria	Exclude Criteria	Refs.
- No previous denture experience, received complete dentures during the study - Detectable microbiological *Candida* spp. levels in oral rinse samples - No clinical symptoms of active candidiasis - Controlled medical status	- Presence of oral candidiasis symptoms - Inability to follow the study procedures - Use of topical or systemic antifungal or antibacterial agents within the previous 60 days - Reported of consumption of probiotics - Gastrointestinal (GIT) disorders, heart disease, or diseases that significantly influence immunity	Elsayes et al. [37]
- Patients with a record at the Usak University Faculty of Dentistry - Provided with both maxillary and mandibular complete dentures - Completed questionnaire data on sex, age, oral health practices (denture cleaning, tongue cleaning), duration of edentulism, and antibiotic intake	- Presence of denture stomatitis, oral infection, or oral cancer - Having a handicap that would impede performing oral health practices - Use of antibiotics within the last 3 months	Evirgen et al. [38]
- Adults with no gender limitation - Diagnosis of oral candidiasis based on clinical symptoms/signs and laboratory tests (smear fungal test and/or saliva fungal culture)	- Presence of chronic mucocutaneous candidiasis or systemic fungal infections - Use of systemic antifungal agents or antibiotics within 1 month prior to participation - Use of topical antifungal agents (except Daktarin or nystatin cream/suppository for vaginal candidiasis) within 2 weeks prior to participation - History of allergy or intolerance to *S. salivarius* K12 lozenges - Pregnant or lactating women - HIV infection - Psychological disorders or other conditions causing uncooperativeness - Abnormal liver or kidney function - Participation in other clinical trials within 1 month prior to enrolment	Hu et al. [39]
- Oral colonization by *Candida* spp. - Absence of clinical signs of denture stomatitis or candidiasis - Patients with common conditions such as hypertension, cardiac diseases, and diabetes were included	- Intolerance to lactose or milk-based foods - Kidney problems - History of head and neck cancer or radiotherapy following this disease - Inability to follow complete study instructions - Use of topical or systemic antifungal or antibacterial agents in the previous 60 days - Refusal to consume cheese daily	Miyazima et al. [40]
- Detectable levels of *Candida* spp. in the palatal mucosa without clinical symptoms of candidiasis - Use of dentures	- Inability to perform or understand the experimental procedures - Use of antifungal agents and/or antiseptic mouth rinses in the previous 6 months - Reported consumption of probiotics - Intolerance to lactose or milk derivatives - Report of severe gastrointestinal disorders or heart disease - Recent transplant recipients - Acquired immune deficiency syndrome (AIDS) - Clinical manifestations of oral candidiasis	Ishikawa et al. [3]
- Nonsmoking elderly individuals - Ability to cooperate with dental examination and saliva sampling - Provided informed consent (both verbal and written)	- Presence of severe chronic diseases or malignancies - Ongoing medication with immunosuppressive or antifungal drugs - Severe HIV dementia or cognitive impairment	Kraft-Bodi et al. [41]
- Presence of atrophic lesion on part or all of the tongue mucosa - Positive detection of *Candida albicans* (>100 Colony-Forming Units, CFU/mL in saliva) - Normal results in physical examinations before medication administration, including: Complete blood cell count, Renal and hepatic clinical chemistry, Urine and stool routine tests, Electrocardiogram, Blood pressure examination, and Abdominal ultrasound and chest X-ray	- Presence of atrophic glossitis - Negative detection of *Candida albicans* or presence of other severe oral mucosal diseases - Wearing complete or partial dentures (due to local microenvironmental effects) - Severe systemic diseases or anemia - Abnormal results in routine physical examinations - Ongoing antibiotic therapy during the trial - Failure to complete return visits or follow-up assessments	Li et al. [20]
- Women were recruited from the Women’s Interagency HIV Study (WIHS) - HIV-infected and HIV-uninfected women were both eligible - Provided written informed consent	- History of hysterectomy - Attempting to become pregnant - Treatment with any antifungal agent at the time of study initiation	Hu et al. [1]
- Clinically healthy status - Institutionalized women aged 65 years or older - No use of antibiotics or immunosuppressive medications in the previous 3 months - Presence of periodontal disease, periodontal pockets, or mucosal lesions at baseline - Provided written informed consent (participant and caregiver)	- Presence of emergency conditions requiring immediate treatment - Emergence of any new factor during the study (e.g., use of antibiotics) that could influence the results	Mendonça et al. [21]
- Independent elderly people - Recruited from old people’s homes and sheltered housing units in the Helsinki area of Finland - Gave written informed consent	- Presence of dementia (mini-mental state (MMS) test of over three points) - Taking current oral yeast medication	Hatakka et al. [19]

**Table 3:** Outcome characteristics of probiotic interventions in oral candidiasis.

Probiotic Microorganisms Used	Probiotic Count (CFU per Unit or Serving)	Delivery Form and Treatment Duration	Administration	Outcome Measurement	Results	Refs.
- *Lactobacilli* strains (*L. reuteri, L. paracasei*, *L. salivarius*, *L. rhamnous*, *L. acidophilus*, *L. plantarum*, and *L. lactis*) - *Bifidobacterium* strains (*B. longum*, *B. animalis*, *B. breve*, *B. bifidum*, *B. infantis*)	- 10^9^ CFU per tablet	- Lozenge - Eight weeks - one lozenge daily (probiotic or placebo)	- The lozenge was dissolved in the mouth after denture removal, with no drink intake for 60 min post-administration	- Quantitative analysis of oral *Candida* load was performed by culturing samples on Sabouraud dextrose agar (SDA) with chloramphenicol and CHROMagar *Candida*; species identification was based on colony morphology and confirmed using VITEK II automated biochemical testing	- Probiotic group showed a significant *Candida* reduction vs. placebo (*p* < 0.001), with 60% negative colonization; effect remained significant 4 weeks post-cessation despite partial relapse.	Elsayes et al. [37]
- *Lactobacillus acidophilus*, *L. rhamnosus*, *L. casei*, and *Bifidobacterium bifidum*	- 5 × 10^9^ CFU/sachet	- Sachet - Ten days	- One sachet dissolved in water and ingested daily	- Microbial counts on palate, cheek, tongue, and denture surfaces were determined by spread plating. Yeasts and Molds on malt agar, Gram-positive bacteria on blood agar, Gram-negative bacteria on eosin methylene blue (EMB), and total mesophilic bacteria on plate count agar (PCA) - Results expressed as CFU/mL based on dilutions	- Significant reduction in microbial counts after probiotic intake (*p* < 0.05) on all oral regions and media, except malt agar on dentures - Greatest reduction on tongue and cheek - Median CFU/mL decreased across PCA, malt, blood agar, and EMB - Regional differences are less pronounced post-intervention	Evirgen et al. [38]
- *Streptococcus salivarius* K12	- Counts ≥ 1 × 10^9^ CFU per lozenge	- Lozenge - four weeks (probiotic or placebo)	- one lozenge of *S. salivarius* K12 twice a day (BID) and a nystatin tablet (500,000 Units) three times a day (TID), both for up to 4 weeks - A placebo lozenge was used in the control group - 1-week post-treatment follow-up	- Mycological Cure: Defined as negative microscopy results using 10% potassium hydroxide (KOH) and absence of *Candida* growth in culture - Clinical Symptoms: Self-reported symptom and lesion scores (0–3) - Treatment Courses: Time until laboratory-confirmed negative tests - Recurrence: Clinical and mycological evaluation 1 week post-treatment - Adverse Events (AEs): Monitored throughout the study based on reported symptoms	- Mycological Cure Rate: Significantly higher in K12 group (90.48%) vs. control (55.56%) (*p* = 0.008) - Overall Cure: No significant difference (*p* = 0.078) - Treatment Duration: Median treatment courses were shorter in the K12 group (3 weeks) compared to the control group (4 weeks) - Clinical Symptoms: No significant difference in remission, both improved post-mycological cures - Adverse Events: No significant difference in safety (*p* > 0.05), no severe events	Hu et al. [39]
- *L. acidophilus* NCFM, *L. rhamnosus* Lr-32	- 7 to 8 log CFU/g	- Probiotic Cheese - 8 weeks received 20 g of fresh white cheese every 2 weeks (experimental groups or control group)	- Experimental groups: T1 (cheese *with Lactobacillus acidophilus* NCFM); T2 (cheese with *Lactobacillus rhamnosus* Lr-32) - Cheese consumption: Daily after denture removal	- Oral *Candida* CFU/mL: Assessed at baseline, week 4, and week 8 via saline mouthwash; cultured on SDA with chloramphenicol. Species identified using CHROMagar, biochemical tests, germ tube, and growth at 42–45 °C and in hypertonic sodium chloride (NaCl) medium	- The mean levels of *Candida* were significantly reduced in experimental groups but not in control (*p* < 0.05)	Miyazima et al. [40]
- *L. rhamnosus* HS111, *L. acidophillus* HS101, *B. bifidum*	- 10^8^ CFU (3.3 × 10^7^ CFU of each) per capsule	- Capsule - Five weeks - One capsule daily (probiotic or placebo)	- The capsule contents were applied to the upper clean maxillary denture palate, which was then used normally to maintain contact with the palatal mucosa	- Quantification of *Candida* load (CFU/mL) from palatal mucosa samples cultured on SDA with chloramphenicol; *Candida* species identified phenotypically (germ tube, microculture, auxanogram, zymogram), with differentiation of *C. albicans* and *C. dubliniensis* via growth in hypertonic broth (6.5% NaCl)	- *Candida* detection was significantly lower in the probiotic group (16.7%) than in the placebo group (92.0%) at the end of the experiment (*p* < 0.0001)	Ishikawa et al. [3]
- *L. reuteri* (DSM 17938 and ATCC PTA 5289)	- 10^8^ CFU total	- Lozenges - Twelve weeks (probiotic or placebo)	- The probiotic or placebo group received 2 lozenges daily, which they were instructed to let slowly melt in the mouth - Their intake was monitored weekly	- Prevalence and amount of oral *Candida* Growth: Assessed using Dentocult CA dip-slide (saliva and pooled plaque cultured on selective Nickerson’s medium with chloramphenicol and gentamicin, incubated at 37 °C for 4 days); CFUs scored 0–3 - Oral Hygiene: Presence/absence of visible plaque - Gingival Inflammation: Bleeding on probing	- Significant reduction in high *Candida* counts (saliva/plaque) in probiotic group (*p* < 0.05)	Kraft-Bodi et al. [41]
- *B. longum*, *L. bulgaricus*, and *S. thermophilus*	- *B. longum* (5 × 10^6^ CFU), *L. bulgaricus* (5 × 10^5^ CFU), and *S. thermophilus* (5 × 10^5^ CFU) per tablet	- Lozenges - Four weeks (probiotic or control group)	- Group A received 2% sodium bicarbonate (30 s gargle), then 2% nystatin paste (10 min later), followed 1 h later by probiotic lozenges (*B. longum, L. bulgaricus, S. thermophilus*) - Group B received only sodium bicarbonate and nystatin - Treatment was applied three times daily	- Pain assessment: Self-reported using a 10 cm Visual Analogue Scale (VAS) (0 = no pain; 10 = extreme pain) - Hyperaemia evaluation: Graded on the lingual dorsum mucosa using a clinically validated four-level red colour card, under standard lighting - *Candida* spp. detection: Saliva and lingual swab samples were 10-fold diluted in phosphate-buffered saline, cultured on both tryptone polypeptone yeast extract agar and CHROMagar Candida, and incubated at 37 °C for 48 h under microaerophilic conditions (80% oxygen, 10% hydrogen, and 10% carbon dioxide) - Other microorganisms: Aerobic/facultative anaerobic cultures were performed on tryptone polypeptone yeast extract agar from the same diluted samples, incubated at 37 °C for 48 h	- *Candida* spp. detection: Significantly reduced in saliva in experimental group compared to control after 4 weeks (8.21% vs. 34.6%, *p* = 0.038). Both groups showed reductions on the dorsum after 2 and 4 weeks (*p* = 0.000) - Pain Level (VAS): Decreased significantly in both groups after 2 and 4 weeks (*p* = 0.000); The experimental group showed greater improvement at 2 weeks (*p* = 0.025) - Hyperaemia: Decreased significantly in both groups after treatment (*p* = 0.000); No significant difference between groups reported	Li et al. [20]
- DanActive™ and YoPlus™ yogurt (containing *Lactobacillus* and *Bifidobacterium* genera)	- CFU not specified (One 3.1-ounce yogurt of DanActive™ per day, One 4-ounce yogurt of YoPlus™ per day)	- Yogurt - 30 days (15 days DanActive™, 15 days YoPlus™) over a 60-day intervention period	- DanActive™ (15 days, one 3.1-ounce yogurt per day) starting on day 60 - Followed by a 30-day washout (no yogurt or probiotic products) - Then YoPlus™ (15 days, one 4-ounce yogurt per day) starting on day 106 - Daily intake and symptoms were recorded in a diary	- Primary outcome: Presence of vaginal and oral fungal colonization, by microscopy (10% KOH) and cultures on SDA, Sabouraud’s dextrose agar with chloramphenicol (SABC), and CHROMagar, followed by species-level identification via germ tube test and chlamydospore assay (for *C. albicans*), and the analytical profile index 20C yeast identification system - Secondary outcomes: Self-reported symptoms and conditions in the past 30 days: constipation, diarrhea, loose stool, vaginal infections, and use of over-the-counter (OTC) medications (present/absent)	- Oral Candida: Slight reduction observed during DanActive™ period (63% → 50%) and YoPlus™ period (58%), but not statistically significant - DanActive™ significantly reduced vaginal colonization (*p* = 0.03) - OTC medicine use significantly reduced (DanActive™ *p* = 0.01; YoPlus™ *p* = 0.02) - Constipation: also significantly reduced in both probiotic periods (DanActive™ *p* = 0.02; YoPlus™ *p* < 0.01)	Hu et al. [1]
- *L. casei*, and *B. breve*	- *Lactobacillus casei*): 2 × 10^7^ to 10^9^ CFU/mL *Bifidobacterium breve*): 5 × 10^7^ to 10^9^ CFU/mL	- Probiotic powder in juice - Thirty days	- One gram (content of one envelope) of the probiotic mixed with juice, given 3 times a week at a fixed hour for 30 days by caregivers	- Unstimulated saliva (2 h post-hygiene) stored at −20 °C with inhibitors - *Candida* CFU/mL by culture on SDA and chloramphenicol (37 °C, 48 h) identification via Gram stain, phenotypic, and biochemical tests - Anti-*Candida* IgA: Measured by ELISA using surface antigens; absorbance read at 450 nm.	- Reduced *Candida* prevalence (92.9% → 85.7%, *p* < 0.05), CFU/mL (*p* = 0.02 Wilcoxon; *p* = 0.00 ANOVA; *p* = 0.03 *t*-test) *, and non-*albicans* spp. - Increased IgA (*p* = 0.00 ANOVA; *p* = 0.01 *t*-test); 61.9% ↑ IgA, 65.4% ↓ *Candida*	Mendonça et al. [21]
- *L. rhamnosus* GG, *L. rhamnosus* LC705, *Propionibacterium freudenreichii* ssp. *shermanii* JS	- 10^7^ CFU (colony-forming units)/g of each of the probiotic strains	- Cheese - Sixteen weeks (probiotic or control)	- Daily 50 g probiotic cheese or control cheese (no probiotics) after a 3-week run-in - Probiotic cheese incorporated *Lactococcus lactis* and *Lactobacillus helveticus* as starter cultures, along with probiotic strains *L. rhamnosus GG*, *L. rhamnosus LC705*, and *P. freudenreichii* ssp. *shermanii JS*. Control cheese used only *L. lactis* as a starter culture	- High salivary yeast prevalence (≥10^4^ CFU/mL) assessed using Dentocult^®^ CA slides (semi-quantitative; score ≥ 2 after 48 h at 37 °C) - Species identification was performed via CHROMagar *Candida* - Salivary flow rates (stimulated/unstimulated) measured over 5 min; buffering capacity assessed by Dentobuff^®^ Strip (classified as low, intermediate, high)	- The probiotic group had reduced yeast counts (adjusted OR = 0.25, *p* = 0.004) - Confirmed by the per-protocol analysis (adjusted OR = 0.23, *p* = 0.01) - Prior use of lactic acid bacteria was linked to lower baseline yeast counts (*p* = 0.03)	Hatakka et al. [19]

* ANOVA and Student’s *t*-test were used for normally distributed data, and the Wilcoxon test was used for data with non-normal distribution. ↑ indicates increase; ↓ indicates decrease.

## Data Availability

No new data were created or analyzed in this study. Data sharing is not applicable to this article.

## Supplementary Materials

The following supporting information can be downloaded at: https://www.mdpi.com/article/10.3390/microorganisms13122883/s1, Table S1: JBI critical appraisal checklist for randomized controlled trials. Table S2: JBI checklist for quasi-experimental studies.

## Abbreviations

The following abbreviations are used in this manuscript:

JBI	Joanna Briggs Institute
CFUs	Colony-Forming Units
CFU/mL	Colony-Forming Units per Milliliter
RCTs	Randomized Controlled Trials
ChiCTR	Chinese Clinical Trial Registry
HIV	Human Immunodeficiency Virus
GIT	Gastrointestinal
AIDS	Acquired Immune Deficiency Syndrome
WIHS	Women’s Interagency HIV Study
MMS	Mini-Mental State test
SDA	Sabouraud Dextrose Agar
spp.	Species
BID	Twice a day
TID	Three times a day
KOH	Potassium hydroxide
NaCl	Sodium chloride
AEs	Adverse Events
VAS	Visual Analogue Scale
ELISA	Enzyme-Linked Immunosorbent Assay
SABC	Sabouraud’s Dextrose Agar with Chloramphenicol
UC	Unclear
