# Supplementary material for: Comparative Effectiveness of Different Probiotic Delivery Methods in Oral Candidiasis: A Systematic Review
Source: Microorganisms. 2025 Dec 18;13(12):2883. doi: 10.3390/microorganisms13122883 (PMC12735766; doi:10.3390/microorganisms13122883)
Supplement: Supplementary file 1 [file microorganisms-13-02883-s001.zip › microorganisms-4029500-supplementary.pdf]

Supplementary Materials: This section presents the supplementary materials. Table S1 displays the JBI Critical Appraisal Checklist for Randomized Controlled Trials, while Table S2 shows the JBI Checklist for Quasi-Experimental Studies.

Table S1: JBI critical appraisal checklist for randomized controlled trials.

Q1	Q2	Q3	Q4	Q5	Q6	Q7	Q8	Q9	Q10	Q11	Q12	Q13	Overall appraisal	Refs.
**Yes**	Yes	Yes	Yes	Yes	Yes	Yes	Yes	Yes	Yes	Yes	Yes	Yes	Include	Elsayes et al. [37]
**Yes**	Yes	Yes	Yes	Yes	Uc	Yes	Yes	Yes	Yes	Yes	Yes	Yes	Include	Hu et al. [39]
**Yes**	UC	Yes	Yes	Yes	Yes	Yes	Yes	Yes	Yes	Yes	Yes	Yes	Include	Miyazima et al. [40]
**Yes**	Yes	Yes	Yes	UC	Yes	Yes	Yes	Yes	Yes	Yes	Yes	Yes	Include	Ishikawa et al. [3]
**Yes**	Yes	Yes	Yes	Yes	No	Yes	Yes	Yes	Yes	Yes	Yes	Yes	Include	Kraft-Bodi et al. [41]
**Yes**	UC	Yes	UC	No	Yes	Yes	Yes	Yes	Yes	Yes	Yes	Yes		Li et al. [20]
**Yes**	Yes	Yes	Yes	Yes	Yes	Yes	Yes	Yes	Yes	Yes	Yes	Yes	Include	Hatakka et al. [19]

Q1. Was true randomization used for assignment of participants to treatment groups? Q2. Was allocation to treatment groups concealed? Q3. Were treatment groups similar at the baseline? Q4. Were participants blind to treatment assignment? Q5. Were those delivering treatment blind to treatment assignment? Q6. Were outcomes assessors blind to treatment assignment? Q7. Were treatment groups treated identically other than the intervention of interest? Q8. Was follow up complete and if not, were differences between groups in terms of their follow up adequately described and analyzed? Q9. Were participants analyzed in the groups to which they were randomized? Q10. Were outcomes measured in the same way for treatment groups? Q11. Were outcomes measured in a reliable way? Q12. Was appropriate statistical analysis used? Q13. Was the trial design appropriate, and any deviations from the standard RCT design (individual randomization, parallel groups) accounted for in the conduct and analysis of the trial?

Table S2: JBI checklist for quasi-experimental studies.

Q1	Q2	Q3	Q4	Q5	Q6	Q7	Q8	Q9	Overall appraisal	Refs
Yes	No	Yes	Yes	Outcome 1: Yes	Outcome 1: Yes	Outcome 1: Yes	Outcome 1<br>Result1: Yes	Outcome 1<br>Result1: Yes	Include	Evirgen et al. [38]
				Outcome 2: Yes	Outcome 2: Yes	Outcome 2: Yes	Outcome 2<br>Result1: Yes	Outcome 2<br>Result1: Yes		
				Outcome 3: Yes	Outcome 3: Yes	Outcome 3: Yes	Outcome 3<br>Result 1: Yes	Outcome 3<br>Result1: Yes		
				Outcome 4: Yes	Outcome 4: Yes	Outcome 4: Yes	Outcome 4:<br>Result1: Yes	Outcome 4:<br>Result1: Yes		
Yes	UC	No	Yes	Outcome1: Yes	Outcome1: Yes	Outcome1: Yes	Outcome1<br>Result1: Yes	Outcome1<br>Result1: Yes	Include	Hu et al. [1]
				Outcome 2: Yes	Outcome 2: Yes	Outcome 2: Yes	Outcome 2<br>Result 1: Yes	Outcome 2<br>Result1: Yes		
				Outcome 3: Yes	Outcome 3: Yes	Outcome 3: Yes	Outcome 3<br>Result1: Yes	Outcome 3<br>Result1: Yes		
Yes	No	Yes	Yes	Outcome 1: Yes	Outcome 1: Yes	Outcome 1: Yes	Outcome 1<br>Result1: Yes	Outcome 1<br>Result1: Yes	Include	Mendonça et al. [21]
				Outcome 2: Yes	Outcome 2: Yes	Outcome 2: Yes	Outcome 2<br>Result1: Yes	Outcome 2<br>Result1: Yes		
				Outcome 3: Yes	Outcome 3: Yes	Outcome 3: Yes	Outcome 3<br>Result1: Yes	Outcome 3<br>Result1: Yes		

Q1. Is it clear in the study what is the "cause" and what is the "effect" (i.e. there is no confusion about which variable comes first)? Q2. Was there a control group? Q3. Were participants included in any comparisons similar? Q4. Were the participants included in any comparisons receiving similar treatment/care, other than the exposure or intervention of interest? Q5. Were there multiple measurements of the outcome, both pre and post the intervention/exposure? Q6. Were the outcomes of participants included in any comparisons measured in the same way? Q7.Were outcomes measured in a reliable way? Q8. Was follow-up complete and if not, were differences between groups in terms of their follow-up adequately described and analyzed? Q9.Was appropriate statistical analysis used?
